# The Role of Programmed Cell Death Ligand-1 (PD-L1/CD274) in the Development of Graft versus Host Disease

**DOI:** 10.1371/journal.pone.0060367

**Published:** 2013-04-04

**Authors:** Heevy Al-Chaqmaqchi, Behnam Sadeghi, Manuchehr Abedi-Valugerdi, Sulaiman Al-Hashmi, Mona Fares, Raoul Kuiper, Joachim Lundahl, Moustapha Hassan, Ali Moshfegh

**Affiliations:** 1 Department of Laboratory Medicine, Division of Clinical Research Center, Experimental Cancer Medicine, Karolinska Institutet, Stockholm, Sweden; 2 Department of Laboratory Medicine, The Unite of Morphologic Phenotype Analysis, Karolinska Institutet and Karolinska University Hospital-Huddinge, Stockholm, Sweden; 3 Department of Clinical Immunology and Transfusion Medicine, Karolinska University Hospital, Stockholm, Sweden; 4 Clinical Research Center (ECM, KFC, Novum), Karolinska University Hospital-Huddinge, Stockholm, Sweden; 5 Department of Oncology and Pathology, Cancer Center Karolinska (CCK), Karolinska University Hospital and Karolinska Institutet, Stockholm, Sweden; Children’s Hospital Boston/Harvard Medical School, United States of America

## Abstract

Programmed cell death ligand-1 (PD-L1/CD274) is an immunomodulatory molecule involved in cancer and complications of bone marrow transplantation, such as graft rejection and graft-versus-host disease. The present study was designed to assess the dynamic expression of this molecule after hematopoietic stem cell transplantation in relation to acute graft-versus-host disease. Female BALB/c mice were conditioned with busulfan and cyclophosphamide and transplanted with either syngeneic or allogeneic (male C57BL/6 mice) bone marrow and splenic cells. The expression of PD-L1 was evaluated at different time points employing qPCR, western blot and immunohistochemistry. Allogeneic- but not syngeneic-transplanted animals exhibited a marked up-regulation of PD-L1 expression in the muscle and kidney, but not the liver, at days 5 and 7 post transplantation. In mice transplanted with allogeneic bone marrow cells, the enhanced expression of PD-L1 was associated with high serum levels of IFNγ and TNFα at corresponding intervals. Our findings demonstrate that PD-L1 is differently induced and expressed after allogeneic transplantation than it is after syngeneic transplantation, and that it is in favor of target rather than non-target organs at the early stages of acute graft-versus-host disease. This is the first study to correlate the dynamics of PD-L1 at the gene-, protein- and activity levels with the early development of acute graft-versus-host disease. Our results suggest that the higher expression of PD-L1 in the muscle and kidney (non-target tissues) plays a protective role in skeletal muscle during acute graft-versus-host disease.

## Introduction

Acute graft-versus-host disease (GVHD) is one of the most disturbing complications following allogeneic hematopoietic stem cell transplantation [1]. It occurs in 40–70% of all patients transplanted with allogeneic stem cells [2], [3]. GVHD develops when alloreactive donor T cells migrate into peripheral tissues. However, the role of different T-cells subsets in GVHD pathogenesis is still controversial [4], [5].

Acute GVHD affects mainly the skin, intestine and liver, while the skeletal muscle, heart, and brain are usually unaffected [6], [7]. Acute GVHD has been proposed to be mediated by Th1-type cells and their inflammatory cytokines including IFN-γ, IL-2 and TNF-α [8], [9], [10]. However, donor T cells lacking IFN-γ still exacerbate GVHD [11]. In contrast, Th2-type cells have been reported to suppress acute GVHD [12].

The CD274 gene is located on mouse chromosome 19 and human chromosome 9 [13]. It encodes a 290 amino acid type I trans-membrane protein designated programmed cell death ligand 1 (PD-L1), a member of the B7/CD28 family [14]. PD-L1 requires interaction with its receptor programmed death 1 (PD-1) for the initiation of the T cell co-inhibitory pathway [15].

Activation of this pathway down-regulates several T cell functions such as proliferation and production of effector cytokines (for example IL-2 and IFN-γ) [16]. On the other hand, blockage of the PD-1/PD-L1 pathway results in enhanced T cell proliferative capacity [17]. This pathway is emerging as a major regulator converting effector T cells into exhausted T-cells during chronic infection and inflammation [18].

Several studies have shown the contribution of PD-L1 to immune regulation and its correlation to cancer [19]. The PD-1/PD-L1 pathway has been established as one of the main mechanisms that terminate the immune response, and it has been proposed to be essential for peripheral tolerance [20], [21]. Moreover, the expression of PD-L1 on non-bone marrow derived cells inhibits T cell effector function in cardiac and pancreatic tissues [22], [23].

Although the role of PD-L1 during transplantation is not fully understood, it has been reported that the PD-1/PD-L1 pathway is involved in suppressing alloreactive T cells that infiltrate the heart in a mouse model of GVHD [24]. Furthermore, we have recently demonstrated that alloreactive T-cells and their inflammatory cytokines are present in differing levels in target versus non-target tissues during the development of GVHD [25]. Taken together, these observations led us to hypothesize that the PD-1/PD-L1 pathway is differentially involved in target as opposed to non-target organs during the development of this disease. The present study was designed to test this hypothesis by monitoring the dynamic expression of PD-L1 in the liver (target organs) and muscle (non-target organ). Our results showed that PD-L1 was highly expressed in non-target organs (muscle and kidney) but expressed to a lower extent in target organs. This might indicate its role in protecting these organs against GVHD.

## Materials and Methods

### Mice

Female BALB/c (H-2K^d^) mice and male C57BL/6 (H-2K^b^) mice 10–14 weeks old were obtained from Scanbur (Sollentuna, Sweden). The mice were kept under pathogen-free conditions in rooms with a 12 hour light/dark cycle. They were fed *ad libitum,* and were allowed to acclimatize for one week before the start of the experiment.

On the designated day, three to six mice per group were sacrificed by cervical dislocation. The mice were perfused through the heart with 20–30 ml ice cold PBS containing 2% FBS and 10 mM EDTA to prevent the effects of circulating inflammatory cells in their tissues. All experiments in this study were approved by the Stockholm Southern Ethics Committee for Animal Research and were in accordance with Swedish Animal Welfare law.

### Bone Marrow Transplantation (BMT)

Bone marrow transplantation was performed as explained previously [25]. Briefly, female BALB/c recipient mice were given busulfan (20 mg/kg/day) for 4 days (days −7, −6, −5 and −4) followed by cyclophosphamide (100 mg/kg/day) for two days (days −3 and day −2). The mice were allowed to rest on day −1, and BMT was performed on day 0. Male C57BL/6 and female BALB/c mice were used as allogeneic and syngeneic donors, respectively. At day 0, bone marrow cells from donor femurs and tibias were flushed and a single cell suspension was prepared. A single cell suspension was also prepared from spleens. The recipient mice were injected with 2×10^7^ of BMC and 3×10^7^ of splenocytes in a volume of 200 µl via the lateral tail vein. This protocol induces acute GVHD in the allogeneic recipient mice with signs of T-cell infiltration into target tissues within seven days post BMT [25].

### Experimental Design

The mice were divided into different groups. A group of untreated and non-transplanted female BALB/c mice were used as controls and are referred to as D-7. A group of mice that were conditioned but not transplanted are referred to as D0. The remaining mice were divided into ten groups depending on the number of days after transplantation and whether they underwent allogeneic or syngeneic (allo, syn) transplantation. They are referred to as D+1allo, D+1syn, D+3allo, D+3syn, D+5allo, D+5syn, D+7allo, D+7syn, D+21allo and D+21syn.

### Tissue Preparation and Total RNA Extraction

Pieces of liver, kidney and skeletal muscle from the thigh were isolated from D-7, D0, D+1allo, D+1syn, D+3allo, D+3syn, D+5allo, D+5syn, D+7allo, D+7syn, D+21allo and D+21syn mice, snap frozen in liquid nitrogen and transferred to −150°C. Total RNA from the frozen tissues was extracted with the RNeasy Mini Kit (Qiagen, Valencia, CA, USA) according to the manufacturer’s instructions. Concentration and integrity of the extracted RNA was confirmed by NanoDrop 1000 (Thermo Scientific, Wilmington, DE, USA) and agarose gel electrophoresis.

### cDNA Synthesis and Real Time PCR (qPCR)

Total RNA (2 µg) was used to synthesize single strand cDNA by using the Superscript II reverse transcriptase system and Oligo(dT)_12–18_ according to the manufacturer’s instructions (Invitrogen Inc., CA, USA). Using cDNA as a template, TaqMan gene expression assays were performed by means of the FAM dye labeling system according to the manufacturer’s instructions (Applied Biosystems, Stockholm, Sweden). TaqMan assays for CD274 were performed as follows: Mm00452054_m1 and the housekeeping gene β actin (ACTB): Mm00607939_s1 were purchased and used for the detection of genes (Applied Biosystems, Stockholm, Sweden). Real time PCR (qPCR) reactions were performed in a total volume of 10 µl using a 384-well plate Thermal Cycler ABI 7900 (Applied Biosystems, Stockholm, Sweden). The results were normalized against the housekeeping gene β-actin by subtracting the Ct value of the housekeeping gene from the Ct value of the specific gene (Ct gene- Ct ACTB), giving a delta Ct (ΔCt) value. Relative gene expression was calculated using the equation 2 ^−ΔCt^. The value of ΔΔCt for an individual gene, when comparing day of interest to D-7, was calculated by subtracting ΔCt of D-7 from ΔCt of CD274 at a specific time point (ΔCt specific day −ΔCt of D-7) and the fold change was calculated using the equation 2 ^−ΔΔCt^.

### Western Blot

10–20 mg frozen samples of muscle, liver and kidney from groups D-7, D0, D+5allo, D+5syn, D+7allo and D+7syn were thawed on ice in 300 µl of Tris NaCl lysis buffer (50 mM Tris-HCl pH 7.4, 150 mM NaCl, 25 mM EDTA, 1 mM NaF, complete mini protease inhibitor cocktail, 1 mM PMSF, 1% Triton×100). In the lysing buffer, the samples were finely minced with a lancet and subjected to interrupted cycles of sonication. Using probe sonication, the instrument was adjusted to a 0.5 cycle and the cycle amplitude to 50 kHz. The samples were subjected to ten 10 second bursts followed by intervals of 30 seconds for cooling, and the procedure was repeated several times until complete lysis was achieved. The lysates were centrifuged at 16 000 rpm for 15 minutes at 4°C. The supernatants were then collected and 150 µl of each was mixed with an equal volume of 2×Laemmli buffer (0.0625 M Tris-HCl pH 6.8, 2% SDS, 10% glycerol and 0.025% bromophenol blue) including 5% 2-mercaptoethanol. The samples and buffer mixtures were boiled at 95°C for 5 minutes. Protein concentrations of the final mixtures were estimated using the 660 nm ® protein assay kit (Pierce, Rockford, IL, USA). 80 µg protein samples were separated using 12% SDS PAGE (sodium dodecyl sulfate polyacrylamide gel electrophoresis) and transferred to nitrocellulose membranes. The membranes were blocked with 5% non-fat dry milk solution for 2 hours at room temperature (RT) and incubated overnight with goat anti-mouse B7-H1 primary antibody (R&D Systems Inc, Minneapolis, MN, USA) at 4°C. After washing, the membranes were incubated with an IRDye ® 800CW conjugated secondary anti-goat antibody (LI-COR, Lincoln, NE, USA) for 1 hour at RT. A rabbit β-actin antibody was used as a loading control. The proteins were visualized using the ODYSSEY imaging system (LI-COR).

### Histology and Immunohistochemistry

Pieces of liver, kidney and skeletal muscle from the thigh were isolated from designated groups (see the section entitled ***Western blot***) and fixed in neutral buffered formalin for 24 hours. They were then transferred to 70% ethanol, dehydrated, and embedded in paraffin according to standard protocol. 4 µm sections were mounted on glass slides and stained with H&E or prepared for immunohistochemistry.

For immunohistochemistry, the sections were treated with 0.3% H_2_O_2_ to block endogenous peroxidase activity after rehydration in a graded alcohol series and antigen retrieval. After blocking with 10% rabbit serum, the samples were incubated overnight with goat anti-mouse PD-L1 (R&D Systems) diluted in 2% bovine serum albumin/phosphate-buffered saline as the primary antibody (Ab). After washing, the sections were incubated with horseradish peroxidase (HRP)-conjugated rabbit anti-goat IgG (ZYMED Lab, Invitrogen) at a dilution of 1∶500. The sections were developed using the HRP-DAB system (R&D Systems), counterstained with Mayer’s hematoxylin (Sigma), dehydrated through a graded alcohol series, and cover slipped.

### Comparison of Gene Expression to that Reported in our Gene Array Paper

For validation, the CD274 expression obtained in this study was compared to the microarray gene expression results in our previous paper [25].

### Measuring IFNγ and TNFα by Sandwich ELISA

At different intervals (D-7, D0, D+5allo, D+5syn, D+7allo, D+7syn), the mice were bled by retro-orbital puncture under light isoflurane anesthesia and thereafter sacrificed by cervical dislocation. The blood samples were centrifuged to obtain serum, which was stored at −80***°***C until analysis. The levels of TNF-α and IFN-γ in the sera were quantified employing commercially available ELISA kits (eBiosciense, San Diego, CA, USA) in accordance with the manufacturer’s instructions.

### Statistical Analysis

Statistical analysis was performed using STATISTICA version 10 (StatSoft, Tulsa, Oklahoma, USA). The expression of CD274 was analyzed in different organs and at different time points for each type of transplantation (allogeneic or syngeneic) using a one way analysis of variance (ANOVA) test followed by post-hoc Tukey’s honest significant difference test (HSD).

## Results

### mRNA Expression of PD-L1 in Muscle, Liver, and Kidney before and after Chemotherapy-based Conditioning

**Figure 1:**
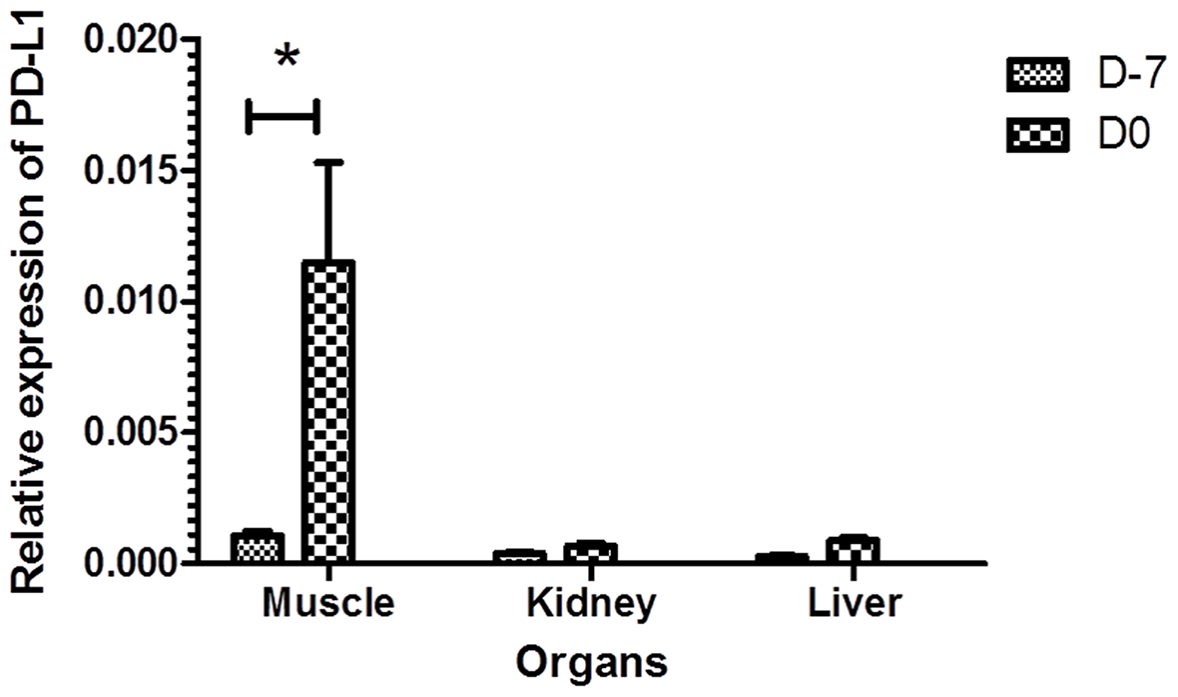
CD274 expression in muscle, kidney and liver in control (D-7) and conditioned mice (D0). Female BALB/c mice were conditioned using a Bu-Cy regimen. Samples from control mice (D-7) and conditioned mice (D0) were collected, mRNA was prepared and qPCR was performed. CD274 expression was calculated as a ratio to the house keeping gene (β actin; ACTB). The results are presented as mean ± SE. * significant difference (p<0.05).

The expression of PD-L1 was significantly higher in the muscle compared to the liver (p<0.05) in untreated animals before conditioning (D-7). Furthermore, the expression of CD274 after conditioning (i.e. at D0) was increased by ten folds in muscle tissue compared to before conditioning (D-7), whereas the increase was minimal in the liver and kidney (Figure 1).

### Expression of PD-L1 in Non-target and Target Organs during the Development of Acute GVHD

**Figure 2:**
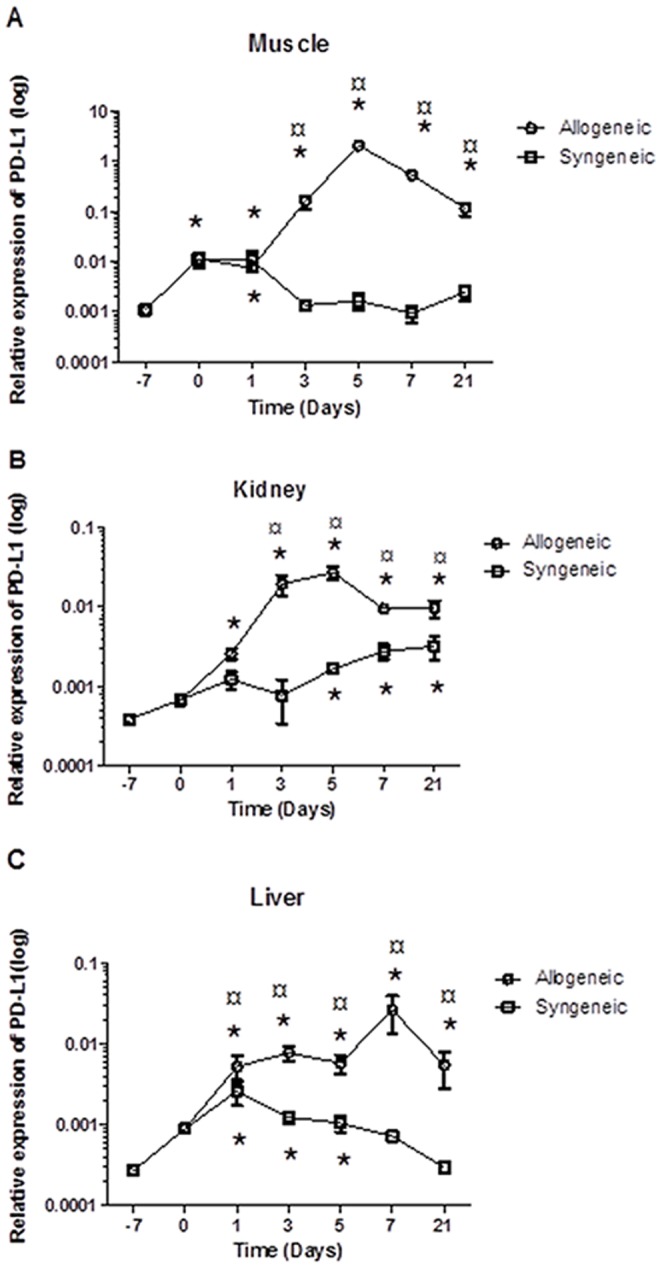
Dynamic expression of CD274 mRNA before and after bone marrow transplantation (syngeneic and allogeneic). Female BALB/c mice were conditioned using a Bu-Cy regimen and transplanted with BM and SP cells from allogeneic or syngeneic donors. Samples from control mice (D-7, before treatment), conditioned mice (D0) and allogeneic- and syngeneic-transplanted mice at different intervals (D+1, D+3, D+5, D+7 and D+21) from the muscle, kidney and liver were collected, mRNA was prepared and qPCR was performed. Normalized (PD-L1) expression was calculated relative to ACTB. The results are presented as mean ± SE. a. Muscle b. Kidney c. Liver *Statistically different (p≤0.05) from the control value (no treatment with Bu-Cy or transplantation, D-7) as determined by the ANOVA test. ¤ Statistically different (p≤0.05) from the syngeneic-transplanted mice as determined by the ANOVA test.

Next, we determined the expression of PD-L1 in different tissues (liver, kidney or muscle) at different time intervals in syngeneic- or allongeneic-transplanted mice. At the time of transplantation (D0) both syngeneic- and allogeneic-transplanted animals exhibited an increase in the expression of PD-L1 in all tested organs (Figure 2a–c). The syngeneic-transplanted group showed a gradual decline in the expression of PD-L1 in all organs, starting on day 1 post transplantation and reaching the same level as controls (D-7) by day +7 (Figure 2a–c). The kidney, however, did not reach the same level as controls until day 21 post transplantation. In contrast, the allogeneic-transplanted group exhibited a continued significant (p<0.05) increase in the expression of this molecule, which peaked at day +5 (muscle and kidney) or +7 (liver) and then gradually declined without reaching control levels even on day 21 post transplantation (Figure 2a–c).

**Figure 3:**
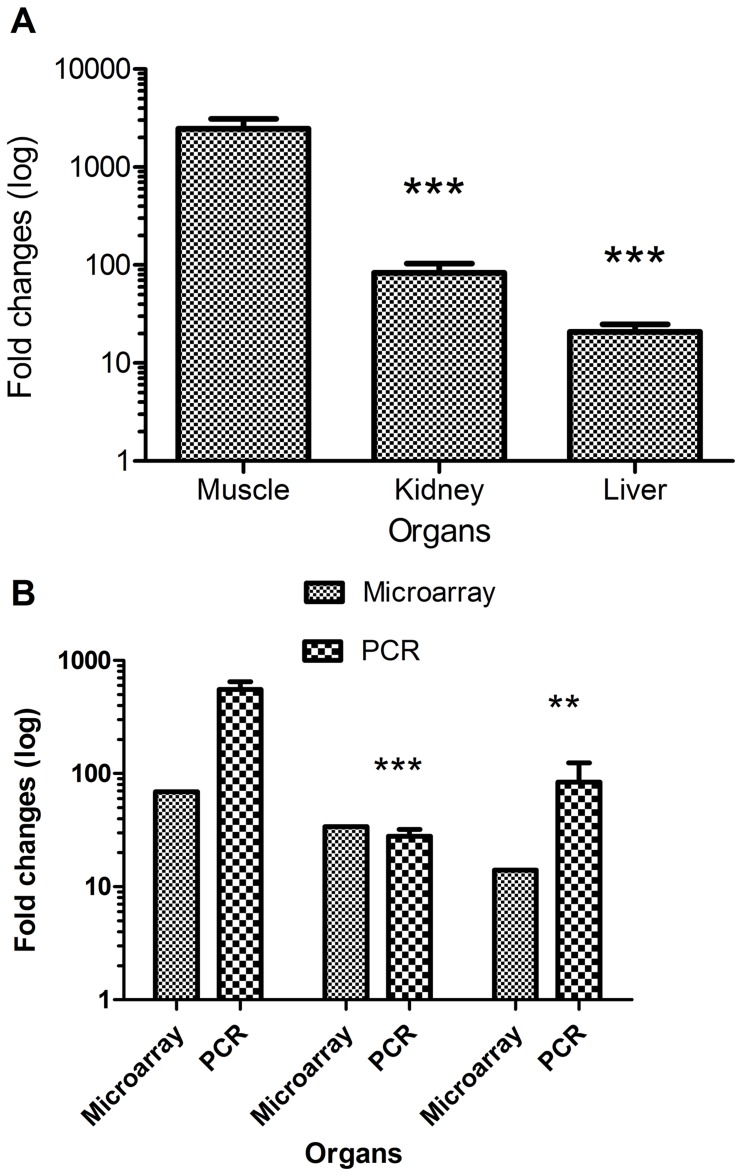
Comparison of mRNA expression fold changes. Fold changes of mRNA expression post allogeneic transplantation relative to control. a. Fold changes of mRNA for muscle and kidney (D+5) and liver (D+7) relative to controls (D-7). The values are presented as mean ± SE. b. Fold changes (D+7) post allogeneic transplantation, compared to microarray data of mRNA fold changes at the same day presented as mean ± SE for PCR results and mean for microarray results (since pooled samples were used). **Significant (p<0.01) ***Significant (p<0.001).

Our abovementioned findings showed that PD-L1 is constitutively expressed in muscle, and that the expression is highly increased after conditioning and allogeneic transplantation. This led us to ask whether PD-L1 plays a role in target and non-target organs involved in acute GVHD. Thus, we next considered the peak day for the relative expression values of PD-L1 in the liver (D+7), kidney (D+5) and muscle (D+5) and transformed them to fold changes compared to its expression in control mice. As shown in Figure 3a, the fold-increase in the expression of PD-L1 in the muscle was significantly higher (p<0.01) than that in the kidney (28-fold) and liver (95-fold). The expression of PD-L1 was still significantly higher (p<0.05) in the muscle than in the kidney and liver 7 days after allogeneic transplantation, as measured by both qPCR and microarray (Figure 3b).

### CD274 at the Protein Level

**Figure 4:**
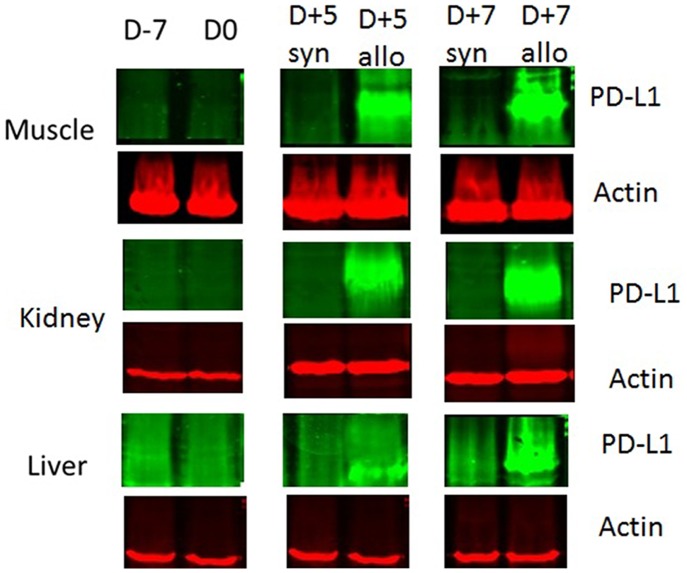
PD-L1 expression at the protein level as determined by western blot. Female BALB/c mice were conditioned using a Bu-Cy regimen and transplanted with bone marrow and spleen cells in an allogeneic or syngeneic setting. Samples were taken from control mice (D-7), conditioned mice (D0) and allogeneic- and syngeneic-transplanted mice at different time points (D+5, D+7). Muscle, kidney and liver were collected, and the lysate was prepared and used for western blot.

Employing western blot technique, we were unable to detect CD274 molecules in tissue samples from syngeneic-transplanted mice at any time point (prior to or after transplantation) (Figure 4). In contrast, mice receiving allogeneic transplants exhibited significantly increased levels of PD-L1 protein in all tested organs 5 days post transplantation. These increased levels were more pronounced in the muscle and kidney and remained at the same level even on day 7 post transplantation (Figure 4).

**Figure 5:**
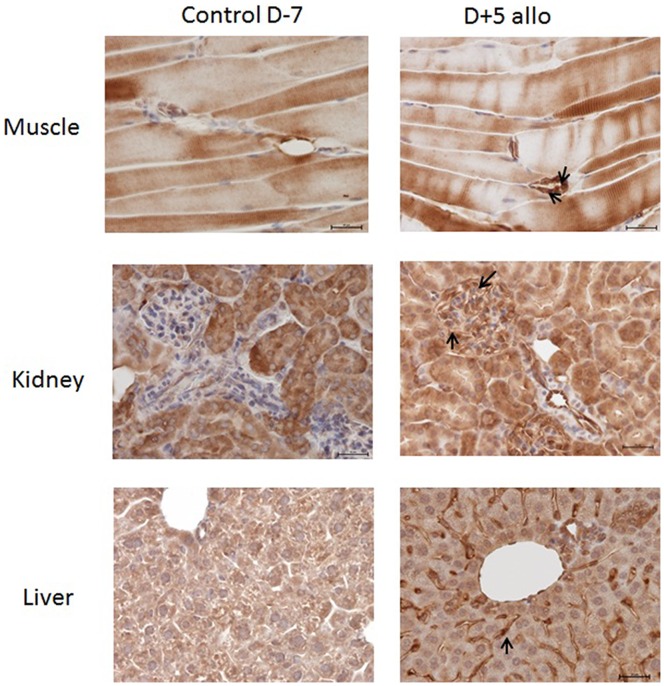
Expression of PD-L1 by immunohistochemistry. Female BALB/c mice were conditioned using a Bu-Cy regimen and transplanted with bone marrow and spleen cells in an allogeneic or syngeneic setting. Samples from control mice (D-7) and from mice five days after allogeneic transplantation (D+5allo) were collected from the muscle, kidney and liver and stained with Anti-B7-H1 Ab. Immunohistochemistry staining for PD-L1 was positive (arrow) after allogeneic transplantation compared to controls (D-7) in all tissues. The strongest PD-L1 staining was obtained in the endothelial cells. Magnification 40X was used in all slides.

The immunohistochemical analysis also demonstrated: a) that the expression of CD274 was up-regulated in the muscle, kidney and liver of transplanted mice 5 days post allogeneic transplantation, and that b) the strongest up-regulation was observed in endothelial cells (Figure 5).

### Production of IFNγ and TNFα during the Development of Acute GVHD

**Figure 6:**
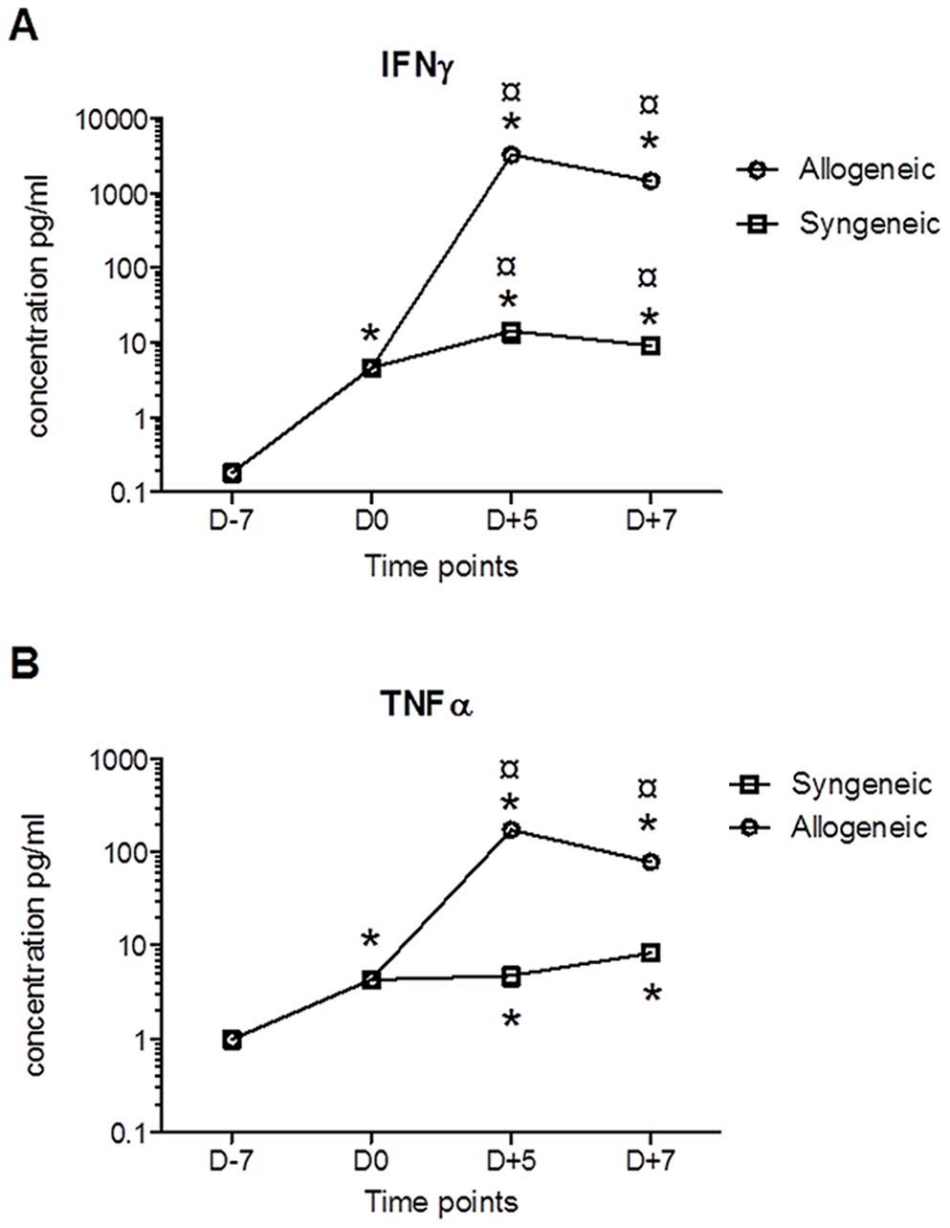
Kinetics of the production of inflammatory cytokines IFNγ and TNFα after chemotherapy based conditioning and transplantation with syngeneic or allogeneic bone marrow and splenic cells. Female BALB/c mice were conditioned using a Bu-Cy regimen and transplanted with bone marrow and spleen cells in an allogeneic or syngeneic setting (see Methods and materials). Blood samples were collected at different time points prior to and after transplantation (D-7, D0, D+5 and D+7), and serum was separated. Thereafter, serum levels of IFNγ and TNFα were measured employing ELISA techniques. *Statistically different (p≤0.05) from the control value (no treatment with Bu-Cy or transplantation, D-7) as determined by the ANOVA test. ¤ Statistically different (p≤0.05) from the syngeneic-transplanted mice as determined by the ANOVA test.

It has been shown that inflammatory cytokines, IFNγ and TNFα in particular, play a crucial role in the development of GVHD [26]. These cytokines are also shown to be involved in the induction of PD-L1 expression [27]. Therefore, we next evaluated the production of IFNγ and TNFα in mice subjected to syngeneic or allogeneic transplantation. As shown in (Figure 6a) and (Figure 6b), serum levels of these cytokines were significantly (p<0.01) enhanced in all treated animals after Bu-Cy chemotherapy (D0). Moreover, the enhancement of IFNγ and TNFα serum levels was further potentiated in allogeneic- but not syngeneic-transplanted mice, exhibiting a peak on day +5 and a slight decline on day 7 post transplantation.

## Discussion

In the present investigation, we studied the dynamic expression of PD-L1 in different organs during the development of murine acute GVHD following transplantation with allogeneic BM and splenic cells.

Our observation that the relative expression of PD-L1 mRNA in the skeletal muscle of untreated control mice (D-7) was higher than that in the liver and kidney strongly suggests that skeletal muscle constitutively expresses more PD-L1. Our next finding, that the mRNA level of PD-L1 is increased in all tested organs upon chemotherapy conditioning, implies that Bu-Cy chemotherapy as the conditioning regimen can per se up-regulate the expression of this ligand. Since conditioning is known to be associated with the cytokine storm that constitutes the first step in acute GVHD development [7], it is also possible that conditioning-induced cytokine storm is responsible for the up-regulation of PD-L1 in different organs. In fact, this possibility is supported by our finding that serum levels of IFNγ and TNFα are significantly increased by conditioning prior to syngeneic or allogeneic transplantation.

In the past few years, different studies have focused on exploring the role of CD274 during HSCT. [24], [28], [29] For instance, it has been shown that during the development of murine GVHD across minor histocompatibility antigen barriers, the expression of PD-L1 is increased in the non-target organ (heart muscle) but not the target organ (intestine) [24]. In line with this finding, we observed the highest expression of PD-L1 in the non-target organ (the muscle) and the lowest expression in the known target organ (the liver). The expression of PD-L1 in the kidney was not as high as in the muscle, but higher than that found in the liver. Previously, our group has shown that the kidney could be a target organ. [25] Relating our results in this investigation to our previous work, we propose that the level of PD-L1 expression may be important in determining whether or not an organ is a target for GVHD.

It is well known that PD-L1 expression and its interaction with PD-1 carry inhibitory signals to activated T-cells [30], [31], [32]. Furthermore, its blockage or absence leads to different diseases, and it regulates T-cell activation as well as contributes to peripheral tolerance [23], [31]. Tanaka et al demonstrate the critical role of PD-L1 in induction and maintenance of peripheral transplantation tolerance, which is due to its ability to alter the balance between pathogenic and regulatory T cells. They also showed that the expression of PD-L1 in donor tissue is critical for prevention of in situ graft pathology and chronic rejection [33]. These facts may further raise the question whether PD-L1 renders the muscle and kidney resistant to acute GVHD or if it merely detects early acute GVHD. The present mouse model of acute GVHD is characterized by major histocompatibility differences between donor and recipient. The recipient mice develop acute GVHD within the first week after transplantation, and the maximum serum levels of IFNγ, TNFα and IL2 are reached at day 5 post transplantation [34], [35]. The high IFNγ is known to induce expression of PD-L1 in different tissues, and synergy with TNFα has been proven to further increase this effect. [27] The high levels of PD-L1 expression in muscle and kidney paralleled reported peak levels of IFNγ, TNFα, and IL2 in the serum. Our data therefore indicate that these immune modulators might contribute to the PD-L1 expression in our model.

Skeletal muscle fibers in humans do not show physiological PD-L1 expression under normal conditions, but they do in inflammatory conditions [27], [36], [37]. Muscle fibers can act as non-professional antigen-presenting cells, which upon activation may express molecules similar to other antigen presenting cells such as MHC (major histocompatibility complex) and PD-L1. In vivo expression of PD-L1 correlates with the presence of inflammatory cells [27]. Thus, inflammation-induced expression of PD-L1 in muscle could act locally as a negative immune-regulator. However, a question of chronicity arises, since it has been found that prolonged PD-L1 expression causes exhaustion of T cells and leads to the development of chronic inflammation [38], [39]. Furthermore, it has been reported that inflammation of the skeletal muscle could be a manifestation of chronic GVHD, [39] which opens the way for further studies.

In conclusion, we propose that an inflammatory environment during HSCT orchestrates the organ-specific expression of PD-L1 and may contribute to relative resistance against acute GVHD in non-target organs. The high expression of PD-L1 observed in the muscle and kidney may prevent or at least postpone its involvement during the acute phase of GVHD. Urgent studies to evaluate PD-L1 as a potential biomarker for GVHD are warranted.
